# Is sentinel lymph node biopsy without frozen section in early stage breast cancer sufficient in accordance with ACOSOG-Z0011? A retrospective review from King Chulalongkorn Memorial Hospital

**DOI:** 10.1186/s12893-022-01709-6

**Published:** 2022-07-06

**Authors:** Nattanan Treeratanapun, Bhoowit Lerttiendamrong, Voranaddha Vacharathit, Kasaya Tantiphlachiva, Phuphat Vongwattanakit, Sopark Manasnayakorn, Mawin Vongsaisuwon

**Affiliations:** Department of Surgery, Faculty of Medicine, King Chulalongkorn Memorial Hospital, Chulalongkorn University, Sirindhorn Building 1873, Rama 4 Rd., Lumphini, Bangkok, 10330 Thailand

**Keywords:** Permanent section (PS), Frozen section (FS), Early-stage breast cancer, Sentinel lymph node biopsy (SLNB), ACOSOG Z0011

## Abstract

**Background:**

In 2021, there is an increased global trend for sending sentinel lymph node biopsy (SLNB) specimens for permanent section (PS) without intraoperative frozen sections (FS). This pilot study conducted in Thailand determines the re-operation rate for SLNB without FS.

**Method:**

We retrospectively reviewed 239 SLNB cases without FS at King Chulalongkorn Memorial Hospital from April 2016 to April 2021. The patients were diagnosed with primary invasive breast cancer with clinically negative nodes. The clinical nodal status was assessed from physical examination. The re-operation rate was determined by the number of positive SLNs; where 3 more nodal metastases were subjected to a second surgical procedure.

**Result:**

Between April 2016 and April 2021, 239 patients who had undergone SLNB in accordance with ACOSOG Z0011 criteria with PS alone was enrolled. A total of 975 SLNs were removed from these 239 patients, with an average of 4.15 nodes per patient. Out of 239 patients, 21 (8.8%) and 6 (2.5%) had metastatic disease in 1 and 2 nodes, respectively. The remaining 212 (88.7%) patients had no nodal metastasis. None of the patients were subjected to a second surgical procedure.

**Conclusion:**

We conclude that the implementation of SLNB with PS analysis alone in patients who satisfy the ACOSOG Z0011 criteria, with a re-operation rate of 0%, does not have outcomes that would be altered by the standard of care additional FS analysis. With ommision of FS analysis, operation cost, operative time and anesthetic side effects are projected to decrease.

## Introduction

Breast cancer is one of the most common types of cancer worldwide with more than 2 million newly diagnosed cases in 2020 [1]. Treatment of choice for nonmetastatic breast cancer was originally surgical resection with axillary lymph node dissection (ALND), with consideration for postoperative radiation [2]. However, sentinel lymph node biopsy (SLNB) has been proposed as a standard diagnostic component in early breast cancer with clinically negative nodes in order to avoid ALND and its associated complications [3, 4]. In 2017, a study conducted by the American College of Surgeons Oncology Group Z0011 (ACOSOG Z0011) reinforced the idea that ALND may not be needed in certain early-stage breast cancer patients. The ACOSOG Z0011 trial concluded that ALND was not always indicated even in patients with one or two positive SLNs. Their inclusion criteria for the randomized controlled trial was female patients with clinical T1 or T2 N0 M0 breast cancer who were treated with SLNB and breast-conserving therapy. No significant difference in the 10 year overall survival for patients who had undergone SLNB and those who had undergone ALND was found [5].

The evaluation of sentinel lymph nodes (SLNs) consisted of 2 methods: intraoperative frozen section (FS) and permanent section (PS) analysis. Conventionally, ALND was recommended when metastatic disease was detected in the SLNs, regardless of the number of positive nodes. Intraoperative FS analyses were previously performed routinely, in order to reduce the need for the subsequent ALND [6–9]. With the recent implementation of the ACOSOG trial, which recommended ALND only when FS revealed more than 2 nodal metastases, coupled with the additional cost and experienced pathologist requirement for the interpretation of FS, significant reduction of FS in SLNB was reported [9, 10]. Research conducted in Korea in 2020 demonstrated that the benefit of FS in early node-negative breast cancer was questionable and that PS alone might be sufficient in these cases [11]. The false negative rate of the intraoperative FS was reported in the range of 10% to 60%, with multiple studies putting the false negative rate between 15 and 20%[9, 12–16]. With regards to the ACOSOG Z0011, a false negative rate of more than 2 nodal metastases on FS, which would necessitate a second surgical procedure, was reported at around 4% [9]. However, the reoperation rate of PS was not reported and such comparison to the reoperative rate when FS is implemented is still unclear.

At King Chulalongkorn Memorial Hospital (KCMH), a tertiary care hospital in Bangkok, Thailand, discordance of surgical techniques of individual surgeons were identified. Some surgeons performed PS alone for SLNBs while others still routinely performed both the intraoperative FS analysis and PS. Notably, the majority of FS procedures performed in the center revealed less than 3 nodal metastases and thus patients did not have an indication for additional ALND.

In terms of treatment costs, additional FS analysis at KCMH costs up to 1160 baht or approximately 37 US dollars per case (using an exchange rate from Bank of Thailand on 29th April 2021) and the cost was higher in private hospitals. We questioned this practice, prompting an in-depth cost–benefit analysis of using conventional FS in early-stage breast cancer and an analysis of whether sending PS alone was sufficient. We conducted a 5-year retrospective review from 2016 to 2021 to confirm that SLNB without FS was not inferior to those with the additional procedure, in patients satisfying the ACOSOG Z0011 criteria. Outcomes were the re-operation rate prevented by each modality. Epidemiological data of the patients was also reported. Noting that the false negative rate of more than 2 lymph nodes on FS was about 4%, we hypothesized that the re-operation rate in our study would be less than 4%. Should this be true, we aim to reduce FS usage on a national scale, which can reduce the average operative cost, operative time, and time under anesthesia without decreasing the quality of treatment.

## Materials and method

In this study, we retrospectively reviewed all SLNB procedures, which were performed at KCMH between April 2016 to April 2021. There were a total of 1,099 cases within the study period. Only patients with early-stage breast cancer (T1 or T2 breast cancer) and clinically node negative status who underwent breast conservation surgery and SLNB without FS were included. Exclusion criteria in our study were based on the ACOSOG Z0011 protocol, threrefore patients with tumors larger than 5 cm (> T2), mastectomy cases, clinically positive nodes, use of preoperative neoadjuvant chemotherapy, positive axillary lymph node from ultrasonographic guided FNA and non-invasive breast cancer were all excluded. SLNBs with intraoperative FS usage were excluded. The clinical nodal status was assessed from physical examination and radiolographic findings on ultrasonography and mammography. Fine needle aspiration (FNA) was performed on all cases with suspicious radiographic findings, all of which were benign.

**Fig. 1:**
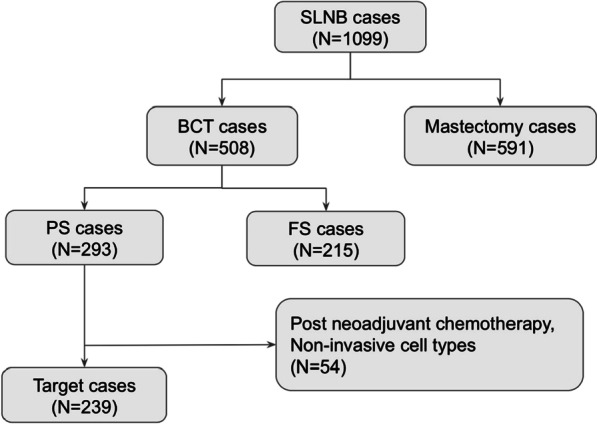
Patient selection flow chart. SLNB: sentinel lymph node biopsy; PS: permanent section; FS: frozen section; BCT: breast-conserving therapy; ALND: axillary node dissection

In 1 case, SLNs could not be identified intraoperatively and was converted to ALND. This patient was excluded from our study. The case was the only instance where SLNB was not successful, accounting for 0.4% of all cases. In total, 239 patients were reviewed in our study (Fig. 1).

We define the re-operation rate as our primary outcome in SLNB operations with PS alone. Re-operation with ALND is indicated in patients with 3 or more nodal metastases, in accordance with ACOSOG Z0011. For each patient, age, laterality, operation type, final pathologic diagnosis, TNM staging classification of the tumor, Nottingham histologic grading, number of SLNs identified by PS, lymphovascular invasion, and HER-2 and hormonal receptor status were reported. Additionally, the clinicopathological status of patients with nodal metastases and those without was compared. Patient consent was not required for this retrospective chart review.

Chest X-rays were routinely performed for preoperative staging in our study in accordance with the NCCN 2022 guidelines. All patients in our study who had undergone breast-conserving therapy received postoperative radiotherapy. The majority of SLNBs in our study were performed using a single-agent mapping tracer (isosulfan blue dye), radioisotope or dual tracer technique. These tracers were injected into breast skin or parenchymal tissue. However, the decision on SLNB identification technique was surgeon-dependent. All pathological diagnoses in this study were based on the serial examination of SLNs using hematoxylin and eosin as the immunohistochemistry staining method.

### Statistical analysis

Data were obtained from the medical records and analyzed using Microsoft Excel version 2019. Statistical analyses were performed using IBM SPSS Statistics ver. 26.0 (IBM Corp., Armonk, NY, USA). The categorical data in this study were described as numbers and percentages. Pearson Chi-square and Fisher exact tests were conducted to compare the categorical variables. Statistical significance was defined as p-value < 0.05 in all variables.

## Results

Between 10th April 2016 and 9thApril 2021, 508 SLNBs were performed in conjunction with breast conserving operations. Intraoperative FS were performed in 215 operations, while the remaining 293 operations had PS alone. A total of 239 out of 293 patients satisfied the ACOSOG Z0011 inclusion criteria and were included in this study. These SLNB cases were stratified by the total number of retrieved SLNs. Three nodes were retrieved in 55 patients, which accounted for the category with the highest number of patients. Twenty-two patients had only 1 retrieved node, however a mininum of 2 SLNs should be retrieved in such scenarios.

Eighty-seven point two percent (208 out of 239) of patients had less than 7 retrieved nodes, while the average number of SLNs retrieved was 4.15 nodes (Table 1). The majority of positive SLNs (24 out of 27) were found in patients in whom 1 to 6 nodes were retrieved. Twenty-one and six patients (8.8%, 2.5%) had 1 and 2 nodal metastases respectively. The remaining 212 (88.7%) patients had no nodal metastases. Since none of the patients had more than 2 nodal metastases, none of the patients were subjected to a second ALND procedure in accordance with the ACOSOG trial Z0011.

**Table 1:** Distribution of retrieved sentinel lymph nodes and presence of metastatic disease

Total SLNs	Patients (N)	No. of patient with no nodal metastasis	No. of patient with 1 nodal metastasis	No. of patient with 2 nodal metastases
1	22	21	1	–
2	45	41	3	1
3	55	48	6	1
4	36	29	5	2
5	24	22	2	–
6	36	32	3	1
> 6	31	29	1	1
975 (4.15)	239	212	21	6

Values are represented as number or number (mean)

SLNs, sentinel lymph nodes

As for the patient demographics, the patient age ranged from 26 to 89 years with a mean and median of 55.4 and 55.0 years respectively. Two hundred and eighteen patients were 40 years of age or older (92.5%). Only 18 patients (7.5%) were under 40 years of age. Comparison of the clinicopathological features between patients with pathological N1 disease and those with pathological N0 disease is demonstrated in Table 2. Out of 131 patients with left-sided breast cancer, 18 patients (13.8%) were found to have a pathological N1 node while the figure stood at less than 10% for the contralateral side. The majority of these cases were ultimately diagnosed with invasive ductal carcinoma which accounted for 195 patients (81.5%). In 10 cases, the outcome of HER-2 immunohistochemistry was equivocal and fluorescence in situ hybridization (FISH) was not performed, thus HER-2 status cannot be confirmed. Radiolographic nodal status was found to be significantly correlated with nodal positivity (p < 0.001). The association between lymphovascular invasion and nodal positivity was found to be statistically significant (p < 0.001). Every patient with evidence of nodal metastasis was found to have invasive ductal carcinoma on histopathology. Most patients without evidence of lymphovascular invasion were found to be negative for nodal metastases (95.5%). However, 46.2% of patients with lymphovascular invasion also had nodal metastases. Patients with pathologic N1 disease had histologic Nottingham grades 1, 2, and 3 at a rate of 2.1%, 13.7%, and 13.6%, respectively. There was a statistically significant correlation between hormonal receptor status and SLN metastasis where 88.6%, 88.3%, and 87.8% of patients were ER positive, PR positive and HER2 positive respectively while also having pathologic N0 nodal status.

**Table 2:** Comparison of Tumor Clinicopathology and Radiolographic nodal status between patients with Pathological nodal status N1 and Pathological nodal status N0

	Total	No. of Patient with Pathological node N1	No. of Patient with Pathological node N0	p-value
Location				0.189
Right	108	9 (8.4%)	99 (91.6%)	
Left	131	18 (13.8%)	113 (86.2%)	
Tumor size				0.095
T1 (< 2 cm)	150	13 (11.9%)	133 (91.1%)	
T2 (2–5 cm)	89	14 (14.8%)	75 (85.2%)	
Radiolographic node				< 0.001*
Borderline	20	11 (55.0%)	9 (45.0%)	
Negative	219	16 (7.4%)	203 (92.6%)	
Histologic grade				0.079
1	48	1 (2.1%)	47 (97.9%)	
2	132	18 (13.7%)	114 (86.3%)	
3	59	8 (13.6%)	51 (86.4%)	
Histopathology				0.333
IDC	195	27 (13.9%)	168 (86.1%)	
ILC	9	0 (0%)	9 (100%)	
DCISM	15	0 (0%)	15 (100%)	
Mucinous	10	0 (0%)	10 (100%)	
Papillary	6	0 (0%)	6 (100%)	
Tubular	1	0 (0%)	1 (100%)	
Mixed	3	0 (0%)	3 (100%)	
Lymphovascular invasion				< 0.001*
Yes	39	18 (46.2%)	21 (53.8%)	
No	200	9 (4.5%)	191 (95.5%)	
Estrogen receptor				0.961
Positive	185	21 (11.4%)	164 (88.6%)	
Negative	54	6 (11.1%)	48 (88.9%)	
Progesterone receptor				0.834
Positive	155	18 (11.7%)	137 (88.3%)	
Negative	84	9 (10.8%)	75 (89.2%)	
HER-2				0.967
Positive	49	6 (12.2%)	43 (87.8%)	
Negative	180	20 (11.1%)	160 (88.9%)	
Not known	10	1 (10.0%)	9 (90.0%)	
Total	239	27	212	

IDC, invasive ductal carcinoma; ILC, invasive lobular carcinoma; DCISM, ductal carcinoma in situ with microinvasion; HER-2, human epidermal growth factor receptor 2

## Discussion

The primary goal of intraoperative FS is to prevent reoperation for ALND. According to the ACOSOG Z0011 trial, an ALND is indicated only when SLNB results in 3 or more nodes positive for metastatic disease [9]. Therefore, intraoperative FS do not provide benefit in patients with only 1 or 2 nodal metastases. Even with routine intraoperative FS, ALND as a second surgical procedure is required if the intraoperative FS was later confirmed to be a false negative. The false negative rate of having more than 2 SLNs positive on FS is still not well defined. However, a study conducted at Imam Khomeini hospital, Iran, revealed a false negative rate of 20.6% when comparing intraoperative FS to PS [8]. Out of those, 4 cases (3.9%) were found with 3 or more diseased nodes. There are also important limitations to the routine practice of sending FS. It is an expensive and a time-consuming procedure which requires an experienced pathologist. Additionally the preparation process could result in irreversible tissue loss which could ultimately alter the final pathological diagnosis. Studies have also found that intraoperative FS was not sufficient to rule out micrometastases [17, 18]. Other studies also recommended the use of PS only in early-stage breast cancer patients who satisfy the ACOSOG trial criteria and discouraged the routine use of intraoperative FS [8, 11, 19].

In this retrospective study, given that ALND is mandatory in patients with at least 3 positive SLNs, we found a reoperation rate of 0% when using PS alone in patients with early-stage breast cancer meeting the ACOSOG Z011 criteria. Previous studies demonstrated that PS alone resulted in a small number of additional ALND (1.9%) [11]. Therefore, the practice of intraoperative FS does not necessarily prevent the second ALND operation compared to SLNB without FS. Three or more SLNs were retrieved in 72.0% of cases with an average of 4.15 nodes per case, which is comparable to the optimal yield of SLNs for SLNB (4 SLNs per case) [20]. When 2 or more SLNs are identified, the false negative rate decreases to an acceptable 5% level as recommended by the American Society of Clinical Oncology (ASCO) guidelines [3, 20]. Breast cancer nomograms have been widely used to predict sentinel lymph node metastasis. One of the first and most validated models is that by Van Zee et al., from the Memorial Sloan-Kettering Cancer Center (MSKCC). The nomogram identified 8 clinicopathological variables that were associated with SLN positivity: age, tumor size, tumor type, tumor location, lymphovascular invasion, multifocality, estrogen receptor status and progesterone receptor status [21]. Other studies supported the MSKCC nomogram findings that age, tumor size, histopathology, estrogen receptor status and progesterone receptor status were valuable predictors of SLN status [22, 23]. A study from Thailand demonstrated that the MSKCC nomogram could accurately predict the probability of SLN metastasis for Thai breast cancer patients, however, only tumor size, histopathology, location, lymphovascular invasion, multifocality and progesterone receptor status were found to be significantly associated with SLNs metastasis [24]. In our descriptive retrospective series, a radiolographically negative nodal status and lack of lymphovascular invasion appear to be negative predictors of lack of SLN metastasis. This finding is consistent with prior studies which found that ultrasound and mammogram findings have a strong predictive value for nodal positivity in early-stage breast cancer with non-palpable axillary nodes [25–27]. Our series suggest that lymphovascular invasion could be a useful predictor that could be added to the breast cancer after further validation. It is notable that HER2 positivity, which represents a highly aggressive tumor subtype, was not associated with nodal metastasis. The inclusion of 10 cases with equivocal HER2 statuses may have altered the statistical outcome. We propose that further research could lead to the integration of lymphovascular invasion presence and radiographic findings into predictive nomograms specific to the Thai breast cancer patient.

Our study demonstrated that in certain well-selected cases, the practice of SLNB with PS alone was not inferior to SLNB with routine FS in terms of reoperation rate. Moreover, in terms of cost-effectiveness, the practice of PS alone could reduce cost of up to 1,160 baht or approximately 37 US dollars per case, which is especially important in the context of low-to-middle income countries (LMICs). Radiolographic nodal status and lymphovascular invasion of the main tumor can be used as predictors of nodal metastasis, which provide a higher nodal positivity prediction compared to other clinicopathology. Our study has a lower percentage of positive nodes after SLNB than that reported elsewhere in the literature. The lower node positivity on SLNB may be attributed to the fact that in 2016 to 2018 time period, surgeons at our center still perform ALND for cases with 1 positive FNA result, as recommended by the 2018 NCCN guidelines and these patients were consequently excluded from the study. However, this practice was changed in the 2019 NCCN guidelines, as now, SLNB can be considered even when FNA results were positive with a few suspicious nodes on imaging. Moreover, our center is a tertiary healthcare center, where strict axillary ultrasound screening is routinely performed, thus more positive nodes were being detected by FNA and these cases with preoperative positive nodes were excluded from our study. This study also included only breast conservative therapy cases, therefore average tumor size is smaller than many other studies (T1 more than 60% of the cases).

However, despite these limitations, this pilot study describes the reoperation rate of SLNB without FS in Thailand. We suggest that such practice is not inferior to the current practice of routine intraoperative FS in patients with early-stage breast cancer and non-palpable axillary nodes. Finally, we encourage a prospective national data collection on tumor clinicopathology and radiolographic nodal status to provide predictive ability, especially in the context of PS utilization alone.

## Conclusions

With a re-operation rate of 0%, we provide a proof of principle to all surgeons in Thailand and low to middle income countries that in patients who satisfy the ACOSOG Z0011 criteria, SLNB with PS alone is sufficient in terms of re-operation prevention. Utilization of PS alone can reduce the operative cost, operative time, and anesthetic side effects from prolonged operations.

## Data Availability

The Datasets generated and/or analyzed in the current study are not publicly available due to the individuals privacy issue but are available from the corresponding author on reasonable request.
